# Half circular modified burr−III distribution, application with different estimation methods

**DOI:** 10.1371/journal.pone.0261901

**Published:** 2022-05-17

**Authors:** Ayesha Iftikhar, Azeem Ali, Muhammad Hanif

**Affiliations:** 1 National College of Business Administration and Economics, Lahore, Pakistan; 2 University of Veterinary and Animal Sciences, Lahore, Pakistan; Government College University Faisalabad, PAKISTAN

## Abstract

The data related to many medical, environmental and ecological variables are often measured in terms of angles wherein its range is defined in [0,*π*). This type of data is referred to as axial or half circular data. Modeling based on half circular data has not received its due share of attention in statistical literature. In this paper, we introduce a new half circular distribution based on inverse stereographic projection technique on modified Burr−III distribution, called the half circular modified Burr−III (hcMB−III) distribution. The basic properties of the proposed distribution are derived. It is common observation that while estimating the parameters of a model, one usually adopts maximum likelihood estimation method as the starting point. In this paper, we consider seven frequentist methods of estimation, besides using maximum likelihood method for estimating the parameters of the hcMB−III distribution. Monte Carlo simulations are performed for investigating the performances of the considered methods in terms of their biases and mean square errors using small, medium and large sample sizes. Finally, one data set related to posterior corneal curvature of the eyes of 23 patients, is analyzed to check potentiality of the newly proposed model.

## Introduction

Circular data analysis is a specific statistical branch that lies somewhere between linear data analysis and spherical data analysis. Circular or directional data is used for measuring observations arising in the fields of meteorology, biology, medical sciences etc. Circular data is measured in degrees and radians. It can also be considered as a point on a circle of unit radius, or a unit vector in a plane. For better comprehension of circular data, it can be regarded as being distributed on the circumference of a unit circle. Circular distributions are of great significance in modeling of cross-bedding data [1], studying paleo-currents [2] measuring wind directions [3], Analysis of time patterns in crime incidence [4], analyzing mother’s day celebrations [5] among various fields. Other significant reviews on circular distributions with their properties can be found in [6–13].

Developing a probability density function for angles has proven to be a challenging assignment for statistician and practitioners. Numerous useful circular models possibly generated by a variety of mechanisms from known probability distributions on the real line or on the plane. A few common methods include:(1) By wrapping a linear distribution around the unit circle (2) through characterizing properties such as maximum entropy (3) an offset method (4) a stereographic projection method that identifies points on the real line with those on the circle circumference.

None of these methods and models concentrate on the semi-circular or the axial data. Sometimes the angular data are given as modulo *π*. Some examples are as follows: (i) the long axis of particles in sediments or the optical axis of a crystal (rather than a direction) (ii) a sea turtle example, where a sea turtle appears from the ocean in quest of a nesting site on dry land (iii) given the angles of initial heading and departure, to trace the debris of aircraft lost problem, semi-circular models are essentially. Thus, we do not require full circular model in such data and is noted by [14], that highlighted this issue and provided some methodology for constructing distributions suitable for modeling these types of data.

There has been little development in the area of half/semi circular distribution. A few more examples of semi-circular data is available in [15]. [16] investigated the semi-circular normal distribution, [17] derived a family of the semi-circular Laplace distributions for modeling semi-circular data by simple projection. [14] constructed some half-circular distributions by applying inverse stereographic projection. Other examples of semi-circular distributions that are generated by inverse stereographic projection can be seen in [18–30].

Motivated by this rationale, In this paper, our objective is to obtain a new distribution, called the half-circular Modified Burr−III distribution (hcMB−III) wherein observations lie on a half circle, i.e., in the range [0, *π*) using the inverse stereographic projection and to derive some of its basic mathematical properties. Next we evaluate and study the behavior of eight different classical estimators for the unknown parameters of the proposed hcMB−III distribution namely, maximum likelihood estimators (MLEs), least-squares estimators (LSEs), weighted least-squares estimators (WLSEs), maximum product of spacings estimators (MPSEs), Cramèr-Von Mises estimators (CVMEs), percentile estimators (PCEs), Anderson-Darling estimators (ADEs) and Right-tail Anderson-Darling estimators (RTADEs). As it is tedious to compare the performances of these estimators theoretically, we conduct extensive simulations for assessing the performances of the said estimators, in terms of their bias and mean squared error(MSE). The novelty of this study is that so far no study has been carried out on hcMB−III distribution or any other half circular distribution using all these estimation methods. A few of the above mentioned researches focus on parameter estimation for derived semi-circular distributions. The focus was primarily given on derivation of trigonometric moments and properties related to trigonometric moments.

The hcMB−III has density that is symmetrical, negatively and positively skewed. The hazard rate of hcMB−III is bathtub and increasing. The flexible nature of the hazard rate function of the hcMB−III distribution will help to serve as the best alternative model to the current models for modeling half-circular real data encountered in diverse fields of life.

The contents of this article are structured as follows. In Section 2, we introduce the hcMB−III distribution and present its cumulative distribution and probability density function (pdf). In the same section, we also present its hazard rate function, sub models of hcM−BIII distribution and uni modality will also be discussed briefly. In Section 3, we discuss various distributional properties like trigonometric moments, characteristics function, skewness and kurtosis of the proposed model. Section 4 demonstrates eight classical methods of estimation to estimate hcMB−III parameters. In Section 5, we perform simulation studies to see the performance of maximum likelihood, maximum product spacings, least squares, weighted least squares, percentiles, Cramèr-von-Mises, Anderson-Darling and Right tailed Anderson-Darling. In Section 6, the usefulness of the hcMB−III distribution is illustrated by using the data of posterior segment of the eyes of 23 patients. Finally, some concluding remarks are given in Section 7.

## The hcMB−III distribution

Several lifetime models have recently been developed and utilized to model data in a variety of fields. A system of twelve kinds of distribution functions based on generating the Pearson differential equation was developed by [31]. The function of density has a variety of forms that are applicable to a wide range of applications [32]. Some recent developments in Burr family of distributions are Burr X Pareto distribution [33], Weibull Burr XII distribution [34], Burr III-Marshal Olkin-G family [35], Unit generalized log Burr XII distribution [36], Unit Burr-XII distribution [37] and Burr XII-moment exponential distribution [38].

The Burr XII distribution is a frequently used variant of the Burr distribution system. Burr−III is the inverse distribution of Burr−XII. For the purpose of statistical modeling, the Burr III distribution has been used in a variety of contexts. For applications of this distribution in various fields one can refer to [39–45]. The cumulative distribution function (cdf) of Burr−III distribution is

$$F(X)={\left\{1+x^{-\beta }\right\}}^{-\alpha },x,\alpha ,\beta >0.$$ (1)

where *α*, *β* are the shape parameters.

In recent past, a new generalization of the Burr−III distribution, called the modified Burr III (MB−III) distribution was proposed by [46]. The cumulative distribution function (cdf) of MB−III distribution is given by

$$F(X)={\left\{1+\gamma x^{-\beta }\right\}}^{-\frac{\alpha }{\gamma }},x,\alpha ,\beta ,\gamma >0.$$ (2)

where *α*, *β*, *γ* are the shape parameters of MB−III distribution.

Modified Burr−III distribution has attracted many researchers due to its tractable properties. [47] studied the transmuted modified Burr III. Characterization of transmuted modified Burr III distribution was done by [48]. [49] developed MBIII-G Family of distributions based on odds ratio of any baseline distribution. The application of Modified Burr III distribution in reliability analysis was done by [50]. [51] originated the McDonald modified Burr–III. [52] developed Cubic rank transmuted modified Burr III-Pareto. Moreover, [53] came up with Unit MB−III distribution. MB−III is a sub model of modified Dagum distribution by [54]. [55] proposed Extended Marshall-Olkin Burr−III distribution.

The Half circular modified burr−III (hcMB−III) distribution can be obtained by applying a transformation *θ* = 2*tan*^−1^(*x*), *θ* *ϵ*(0, *π*). Let m(*θ*) = tan (*θ*/2). By using inverse stereographic projection, the pdf of the hcMB−III distribution is given by *g*(*θ*) = |*m*′(*θ*)|*f*[*m*(*θ*)].

we have

$$|m^{{}^{\prime }}(\theta )|=|\frac{1}{2}sec^{2}(\theta /2)|=\frac{1}{1+cos(\theta )}$$

and

$$f(m(\theta ))=\alpha \beta {\left\{\text{tan}\left(\frac{\theta }{2}\right)\right\}}^{-\beta -1}{\left\{1+\gamma {\left(\text{tan}\left(\frac{\theta }{2}\right)\right)}^{-\beta }\right\}}^{-\frac{\alpha }{\gamma }-1}$$

Consequently, the pdf of hcMB−III (*α*, *β*, *γ*) is given by

$$\begin{array}{c} g\left(\theta \right)=\frac{\alpha \beta }{2}{\text{sec}}^{2}(\frac{\theta }{2}){\left\{\text{tan}(\frac{\theta }{2})\right\}}^{-\beta -1}{\left\{1+\gamma {\left(\text{tan}(\frac{\theta }{2})\right)}^{-\beta }\right\}}^{-\frac{\alpha }{\gamma }-1},0<\theta <\pi . \end{array}$$ (3)

The cdf of hcMB−III distribution is given as

$$\begin{array}{c} G\left(\theta \right)=P(\Theta \leq \theta )=P(2{\text{tan}}^{-1}X\leq \theta ) \end{array}$$

$$\begin{array}{c} =P(X\leq 2{\text{tan}}^{-1}({}^{\theta }/{}_{2}))=\int_{0}^{\text{tan}({}^{\theta }/{}_{2})}F(x)dx \end{array}$$

$$\begin{array}{c} G\left(\theta \right)={\left\{1+\gamma {\left(\text{tan}(\frac{\theta }{2})\right)}^{-\beta }\right\}}^{-\frac{\alpha }{\gamma }} \end{array}$$ (4)

**Fig 1:**
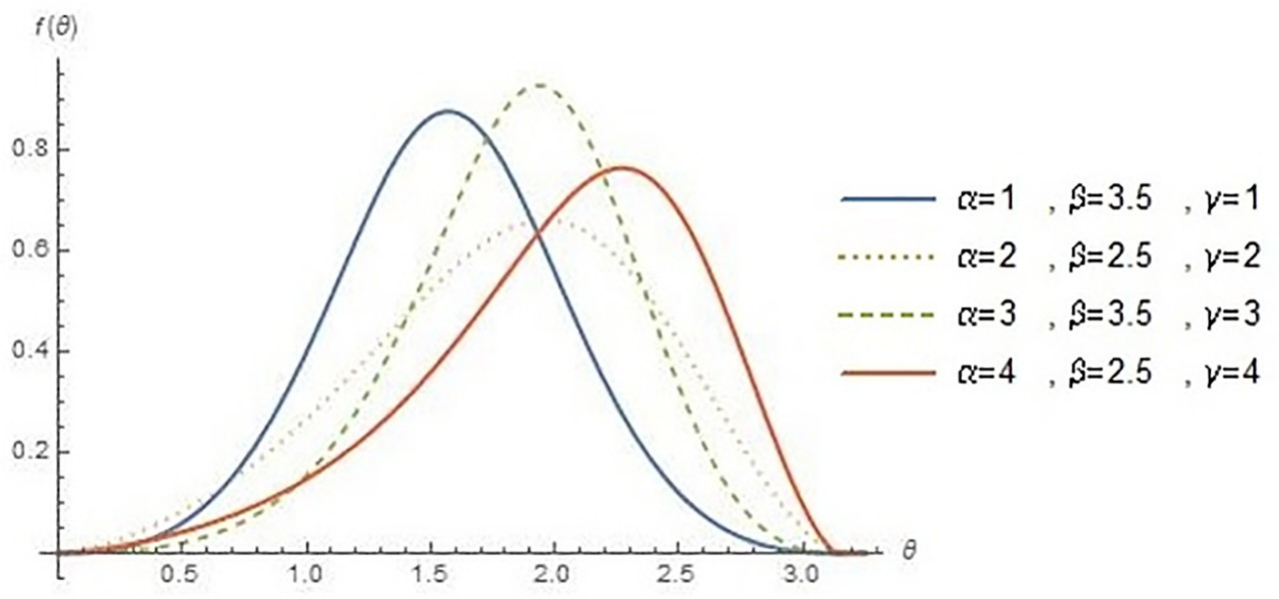
Linear presentation of hcMB−III density.

**Fig 2:**
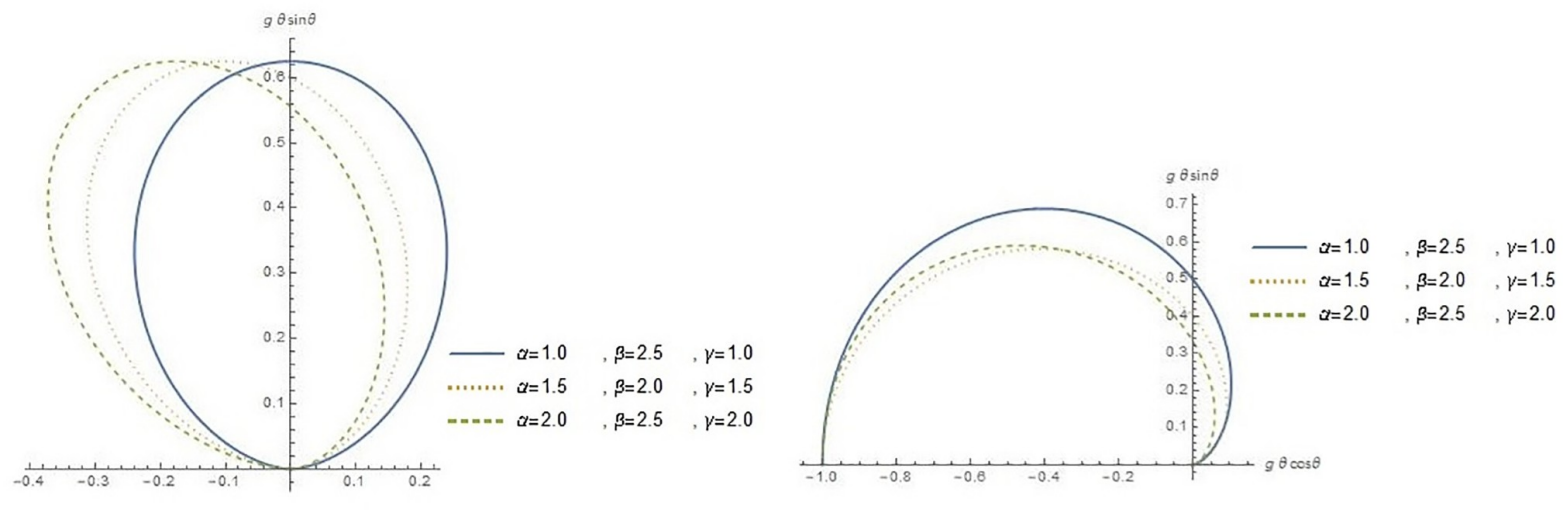
Circular presentation of hcMB−III distribution with its cdf.

Since we do not decide shapes of the density and hazard rate function analytically, we plot them based on some selected parameters value to see their possible shapes. The shape of hcMB−III distribution for various values of (*α*, *β*, *γ*) are presented in Figs 1 and 2 demonstrates circular presentation of hcMB−III distribution and it’s cumulative distribution function. Different values of parameters show the flexibility of hcMB−III distribution such as negatively skewed, symmetric and positively skewed. Therefore, the hcMB−III distribution is quite flexible and can be applied to various data sets.

Consider $z_{\theta }=1+\gamma {\left(\text{tan}\left(\frac{\theta }{2}\right)\right)}^{-\beta }$ and the hazard function of hcMB−III distribution is defined as

$$\begin{array}{c} h\left(\theta \right)=\frac{\alpha \beta {\text{sec}}^{2}(\frac{\theta }{2}){\left(\text{tan}(\frac{\theta }{2})\right)}^{-\beta -1}z_{\theta }^{-\frac{\alpha }{\gamma }-1}}{2(1-z_{\theta }^{-\frac{\alpha }{\gamma }})} \end{array}$$ (5)

**Fig 3:**
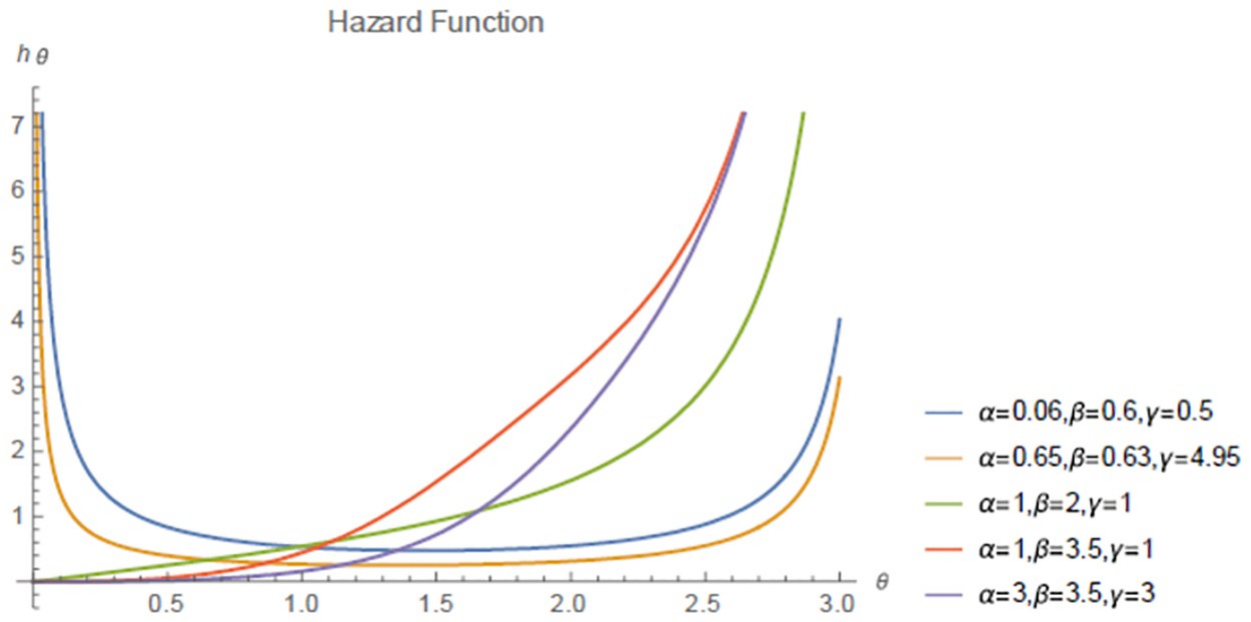
Plot of the hcMB−III hazard rate.

The hazard function of hcMB−III distribution for some parametric values are given below in Fig 3 shows that failure rate function can be increasing and bathtub shaped. Therefore, hcMB−III distribution can be applied to various data sets.

Three sub models of modified burr III distribution were listed by [46]. Hence, hcMB−III also have three sub models named as hc-Generalized Inverse Weibull (hc-GIW), hc-Burr III distribution and hc-Log Logistic (hc-LL) distribution. The cdfs of sub models of proposed model are listed in Table 1 along with their hazard functions.

**Table 1:** Cdfs and hazard functions of the sub models of hcMB−III distribution.

Model	*F*(*θ*)	*h*(*θ*)
hc-Burr III	${\left\{1+{\left(\text{tan}\left(\frac{\theta }{2}\right)\right)}^{-\beta }\right\}}^{-\alpha }$	$\frac{\alpha \beta }{1+\text{cos}\left(\theta \right)}.\frac{{\left\{\text{tan}\left(\frac{\theta }{2}\right)\right\}}^{-\left(\beta +1\right)}{\left\{1+{\left(\text{tan}\left(\frac{\theta }{2}\right)\right)}^{-\beta }\right\}}^{-1}}{{\left\{1+{\left(\text{tan}\left(\frac{\theta }{2}\right)\right)}^{-\beta }\right\}}^{\alpha }-1}$
hc-GIW	$\text{exp}\left\{-\gamma {\left(\alpha {\left\{\text{tan}\left(\frac{\theta }{2}\right)\right\}}^{-1}\right)}^{\beta }\right\}$	$\frac{\gamma \beta \alpha^{\beta }{\left\{\text{tan}\left(\frac{\theta }{2}\right)\right\}}^{-\beta -1}\text{exp}\left[-\gamma {\left\{\alpha {\left(\text{tan}\left(\frac{\theta }{2}\right)\right)}^{-1}\right\}}^{-\beta }\right]}{\left(1+\text{cos}\left(\theta \right)\right)\left\{1-\text{exp}\left[-\gamma {\left\{\alpha {\left(\text{tan}\left(\frac{\theta }{2}\right)\right)}^{-1}\right\}}^{-\beta }\right]\right\}}$
hc-Log Logistic	$\frac{1}{1+\alpha^{\beta }{\left\{\text{tan}\left(\frac{\theta }{2}\right)\right\}}^{{}^{-\beta }}}$	$\frac{\beta \alpha^{-2\beta }{\left\{\text{tan}\left(\frac{\theta }{2}\right)\right\}}^{-1}}{\left(1+\text{cos}\left(\theta \right)\right)\left\{1+\alpha^{\beta }{\left(\text{tan}\left(\frac{\theta }{2}\right)\right)}^{-\beta }\right\}}$

Mode of hcMB−III distribution is derived by taking log of the probability density of hcMB−III distribution.

$$\begin{array}{c} M=log[\frac{\alpha \beta }{2}{\text{sec}}^{2}(\frac{\theta }{2}){\left\{\text{tan}(\frac{\theta }{2})\right\}}^{-\beta -1}{\left\{1+\gamma {\left(\text{tan}(\frac{\theta }{2})\right)}^{-\beta }\right\}}^{-\frac{\alpha }{\gamma }-1} \end{array}$$ (6)

and to find mode put

$$\begin{array}{c} \frac{\partial M}{\partial \theta }=-(\beta +sec(\theta ))cosec(\theta )+[\frac{\beta (\alpha +\gamma )cosec(\theta )}{\gamma +{\left(tan(\frac{\theta }{2})\right)}^{\beta }}]=0 \end{array}$$ (7)

Since it is apparent that the equation has not an explicit solution in the general case. Consequently, we discuss it empirically:

1. ∀ *γ* distribution is bimodal as *α*, *β* → 0.
2. for 0.82 < *β* < 1.32 distribution is uni modal and bimodal other wise as *α*, *γ* → 0.
3. for *α* < 79 distribution is uni modal and bi modal other wise as *β*, *γ* → 0.

### Characteristics function and properties related to trigonometric moments of hcMB−III distribution

The characteristic function of a half/semi-circular model with pdf *g*(*θ*) is defined as

$$\begin{array}{c} \phi \left(\theta \right)=E\left[e^{ip\theta }\right]=\int_{0}^{\pi }e^{ip\theta }g\left(\theta \right)d\theta ,p\in Z \end{array}$$ (8)

$$\begin{array}{c} \phi \left(\theta \right)=\frac{\alpha \beta }{2}\int_{0}^{\pi }e^{ip\theta }{\text{sec}}^{2}(\frac{\theta }{2}){\left\{\text{tan}(\frac{\theta }{2})\right\}}^{-\beta -1}{\left\{1+\gamma {\left(\text{tan}(\frac{\theta }{2})\right)}^{-\beta }\right\}}^{-\frac{\alpha }{\gamma }-1}d\theta \end{array}$$ (9)

**Fig 4:**
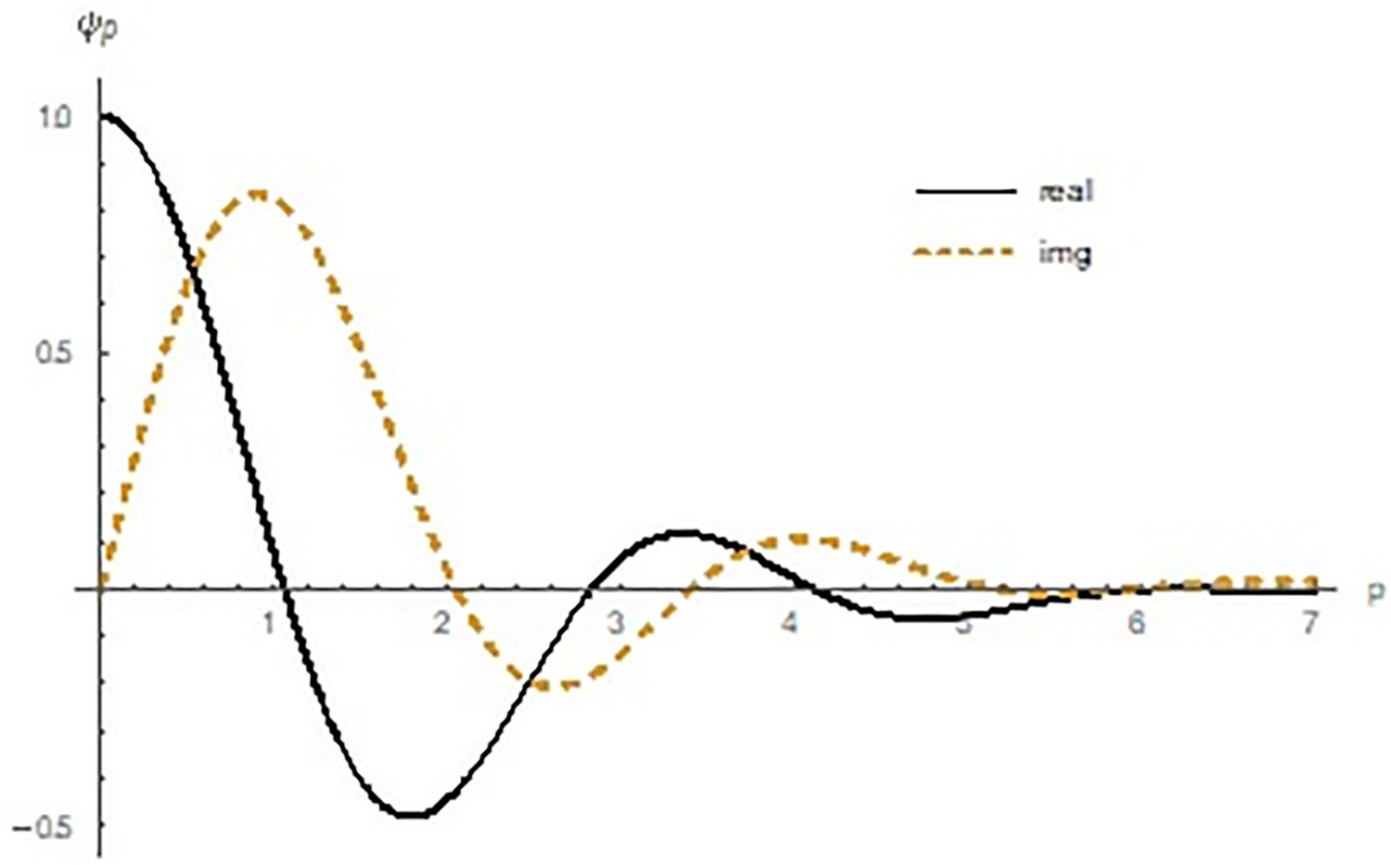
Graphical display of real and imaginary parts of characteristics function of hcMB−III distribution.

The characteristic function defined above also called the pth trigonometric moment. Since *θ* and *θ* + 2*π* represents the same direction so it is necessary to restrict p to integer value. Characteristics function of hcMB−III distribution is presented graphically in Fig 4 for *α* = 1.5, *β* = 3.5, *γ* = 3.5 is as follows:

The trigonometric moments of the distribution are given by *ϕ*; ±1, ±2, ±3, ⋯, where

$$\begin{array}{c} \phi_{p}=\alpha_{p}+i\beta_{p} \end{array}$$ (10)

and

$$\begin{array}{c} \alpha_{p}=E\left[cos\left(p\theta \right)\right]=\int_{0}^{\pi }cos\left(p\theta \right)g\left(\theta \right)d\theta \end{array}$$ (11)

and

$$\begin{array}{c} \beta_{p}=E\left[sin\left(p\theta \right)\right]=\int_{0}^{\pi }sin\left(p\theta \right)g\left(\theta \right)d\theta \end{array}$$ (12)

being the *p*^*th*^ order cosine and sine moments of the random angle *θ*, respectively and are required to study distribution characteristics.

and

*α*_−*p*_ = *E*[*cos*(−*pθ*)] = *E*[*cos*(*pθ*)] = *α*_*p*_
*β*_−*p*_ = *E*[*sin*(−*pθ*)] = −*E*[*sin*(*pθ*)] = −*β*_*p*_
∣*α*_*p*_∣≤ 1, ∣*β*_*p*_∣≤ 1.

Putting *p* = 1, we get 1st order trigonometric moments as

$$\begin{array}{c} \alpha_{1}=\frac{\alpha \beta }{2}\int_{0}^{\pi }cos(\theta ){\text{sec}}^{2}(\frac{\theta }{2}){\left\{tan(\frac{\theta }{2})\right\}}^{-\beta -1}{\left\{1+\gamma {\left[tan(\frac{\theta }{2})\right]}^{-\beta }\right\}}^{-\frac{\alpha }{\gamma }-1}d\theta \end{array}$$ (13)

$$\begin{array}{c} \beta_{1}=\frac{\alpha \beta }{2}\int_{0}^{\pi }sin(\theta ){\text{sec}}^{2}(\frac{\theta }{2}){\left\{tan(\frac{\theta }{2})\right\}}^{-\beta -1}{\left\{1+\gamma {\left[tan(\frac{\theta }{2})\right]}^{-\beta }\right\}}^{-\frac{\alpha }{\gamma }-1}d\theta \end{array}$$ (14)

The direction *μ* is called the mean direction; the mean direction of hcMB−III distribution is defined as

$$\begin{array}{c} \mu =tan^{-1}(\frac{\beta_{1}}{\alpha_{1}}) \end{array}$$ (15)

*α*_1_ and *β*_1_ are defined in Eqs (13) and (14). The mean resultant length (MRL) of hcMB−III distribution is defined as and denoted by *ρ*

$$\begin{array}{c} \rho =\sqrt{\alpha_{1}^{2}+\beta_{1}^{2}} \end{array}$$ (16)

*α*_1_ and *β*_1_ are defined above and mean resultant length (MRL) is invariant under rotation.

A useful measure of dispersion on the circle is the circular variance. Circular variance of hcMB−III distribution is defined as

$$\begin{array}{c} \upsilon =1-\rho =1-\sqrt{\alpha_{1}^{2}+\beta_{1}^{2}} \end{array}$$ (17)

Where 0 ≤ *υ* ≤ 1, *ρ* denotes the MRL and *α*_1_ and *β*_1_ are defined above.

Circular standard deviation of hcMB-III distribution is defined as

$$\sigma =\sqrt{-\text{log}(\alpha_{1}^{2}+\beta_{1}^{2})}$$ (18)

*α*_1_ and *β*_1_ are defined above and circular standard deviation measures the average direction from mean direction.

Circular skewness of hcMB-III distribution is defined as

$$\gamma_{1}=\frac{\beta_{2}^{*}}{{\left(1-\rho \right)}^{\frac{3}{2}}}$$ (19)

$\beta_{2}^{*}$ is 2^*nd*^ trigonometric moment about mean and circular kurtosis measures the kurtosis of circular distribution.

Circular kurtosis of hcMB-III distribution is defined as

$$\gamma_{2}=\frac{\alpha_{2}^{*}-\rho^{4}}{{\left(1-\rho \right)}^{2}}$$ (20)

$\alpha_{2}^{*}$ is 2^*nd*^ trigonometric moment about mean and circular kurtosis measures the kurtosis of circular distribution.

Using expressions in [9, 56] and the first two trigonometric moments, the characteristics of stereographic hcMB−III distribution presented above are calculated numerically and are presented in the S1 Appendix, by using Mathematica 12.0 for some parametric values of hcMB−III distribution. Following results are obtained.

(**i**) Circular measures for fixed *α* and *β* increase in *γ* the mean direction becomes positive.The resultant length is close to 0.5. Values of skewness and kurtosis show that the hcMB− III distribution is positively skewed and platykurtic.
(**ii**) Circular measures for fixed *α* and *γ* increase in *β* the mean direction constant (zero). The resultant length rapidly increases as *β* increases. Values of skewness and Kurtosis show that the hcMB− III distribution is symmetric and platykurtic.
(**iii**) Circular measures for fixed *β* and *γ* increase in *α* the mean direction decreases. The resultant length is close to 1. Values of skewness and kurtosis shows that the hcMB− III distribution is positively skewed and platykurtic.

## Parameter estimation of hcMB−III distribution

In this section, eight different estimation methods are used to estimate the unknown parameters of the hcMB− distribution, such as the maximum likelihood (ML), ordinary least square (OLS), weighted least square (WLS), percentile (PC), maximum product spacing (MPS), Cramer-von-Mises (CVM), Anderson-Darling (AD) and Right-tail Anderson Darling (RTAD). we compare their performance on the basis of simulated samples from the hcMB−III distribution. The details are as followings.

### Maximum likelihood estimates

The method of maximum likelihood is the most frequently used method of parameter estimation. The method’s success stems no doubt from its many desirable properties including consistency, asymptotic efficiency, invariance and simply its intuitive appeal. The log-likelihood function for the vector of parameters *ζ* = (*α*, *β*, *γ*) of the hcMB−III distribution is

$$\begin{array}{c} \ell (\zeta )=n\;\text{log}\;\alpha +n\;\text{log}\;\beta -n\;\text{log}\;2+2\sum_{i=1}^{n}\text{log}[sec(\frac{\theta_{i}}{2})]+\\ \left(-\beta -1)\sum_{i=1}^{n}\text{log}[tan(\frac{\theta_{i}}{2})]+(\frac{-\alpha }{\gamma }-1)\sum_{i=1}^{n}[1+\gamma {\left\{tan(\frac{\theta_{i}}{2})\right\}}^{-\beta }\right] \end{array}$$ (21)

The resulting partial derivatives of the Eq (21) are:

$$\begin{array}{c} \frac{\partial L}{\partial \alpha }=\frac{n}{\alpha }-\frac{1}{\gamma }\sum_{i=1}^{n}\text{log}[1+\gamma {\left\{tan(\frac{\theta_{i}}{2})\right\}}^{-\beta }] \end{array}$$ (22)

$$\begin{array}{c} \frac{\partial L}{\partial \beta }=\frac{n}{\beta }-\sum_{i=1}^{n}\text{log}\{tan(\frac{\theta_{i}}{2})\}+\left(\alpha +\gamma \right)\sum_{i=1}^{n}\frac{{\left\{tan(\frac{\theta_{i}}{2})\right\}}^{-\beta }\text{log}\{tan(\frac{\theta_{i}}{2})\}}{1+\gamma {\left\{tan(\frac{\theta_{i}}{2})\right\}}^{-\beta }} \end{array}$$ (23)

and

$$\begin{array}{c} \frac{\partial L}{\partial \gamma }=\frac{\alpha }{\gamma^{2}}\sum_{i=1}^{n}\text{log}[1+\gamma {\left\{tan(\frac{\theta_{i}}{2})\right\}}^{-\beta }]+(\frac{-\alpha }{\gamma }-1)\sum_{i=1}^{n}\frac{{\left\{tan(\frac{\theta_{i}}{2})\right\}}^{-\beta }}{1+\gamma {\left\{tan(\frac{\theta_{i}}{2})\right\}}^{-\beta }} \end{array}$$ (24)

The MLEs of unknown parameters cannot be derived analytically from the above normal equations because of convoluted non-linear expressions. Therefore, the iterative methods can be used to obtain the estimated values of the unknown *α*, *β* and *γ* simultaneously.

Under some regularity conditions for unknown parameters in the interior of parameter space but not on the boundaries, the asymptotic distribution of $\sqrt{n}\left(\hat{\psi }-\psi \right)$, where *ψ* = (*α*, *β*, *γ*)^*t*^, follows multivariate normal with mean vector zero and variance-covariance matrix is *K*^−1^(*ψ*) i.e. $\sqrt{n}\left(\hat{\psi }-\psi \right)∼N_{3}\left(0,K^{-1}\left(\psi \right)\right)$ where *K*(*ψ*) = *E*[*J*(*ψ*)]. It can be noted that $K\left(\psi \right)=\underset{n\to \infty }{\text{lim}}n^{-1}J\left(\psi \right)$ is the unit information matrix. In fact, 100(1 − λ)% asymptotic confidence interval (ACI) for each unknown parameter can be obtained by using $ACI_{i}=\hat{\psi }\pm z_{\frac{\lambda }{2}}\sqrt{{\hat{J}}^{\psi_{i}\psi_{i}}}$ where ${\hat{J}}^{\psi_{i}\psi_{i}}$ represents the (*i*, *i*) diagonal element of $J^{-1}\left(\hat{\psi }\right)$ for *i* = 1, 2, 3 and $z_{\frac{\lambda }{2}}$ is the quantile $1-\frac{\lambda }{2}$ of the standard normal distribution.

### Ordinary and weighted least square estimates

The least square estimators (LSE) and weighted least square estimators (WLSE) were proposed by [57] to estimate the parameters of Beta distributions. Suppose *F*(*x*_(*i*)_)denotes the distribution function of the ordered random variables *X*_(1)_ < *X*_(2)_ <…< *X*_(*n*)_ be ordered sample of size n from hcMB-III distribution. Then, the expectation of the empirical cumulative distribution function is defined as

$$\begin{array}{c} E[F(x_{\left(i\right)})]=\frac{i}{n+1};i=1,2,\ldots ,n. \end{array}$$

The least square estimates (LSEs) say, ${\hat{\alpha }}_{LSE}$, ${\hat{\beta }}_{LSE}$, ${\hat{\gamma }}_{LSE}$, of *α*, *β* and *γ* are obtained by minimizing

$$\begin{array}{c} QLSE(\zeta )=\sum_{i=1}^{n}{\left(F(x_{\left(i\right)})-\frac{i}{n+1}\right)}^{2} \end{array}$$ (25)

The variance of the empirical cumulative distribution function is defined as

$$\begin{array}{c} V[F(x_{\left(i\right)})]=\frac{i(n-i+1)}{(n+2){\left(n+1\right)}^{2}};i=1,2,\ldots ,n. \end{array}$$

Thus, the weighted least square estimates (WLSEs) say, ${\hat{\alpha }}_{MPS}$, ${\hat{\beta }}_{MPS}$, ${\hat{\gamma }}_{MPS}$, of *α*,*β* and *γ* are obtained by minimizing by minimizing

$$\begin{array}{c} QWLSE(\zeta )=\sum_{i=1}^{n}\frac{{\left(F(x_{\left(i\right)})-\frac{i}{n+1}\right)}^{2}}{V[F(x_{\left(i\right)})]}. \end{array}$$ (26)

### Percentile estimates (PCE)

If the data come from a distribution function which has a closed form, then we can estimate the unknown parameters by fitting straight line to the theoretical points obtained from the distribution function and the sample percentile points. This method was originally suggested by [58, 59] and it has been used for weibull distribution and for generalized exponential distribution. In this paper, we apply the same technique for the hcMB−III(*α*, *β*, *γ*) distribution.

Let *X*_(*i*)_ be the *i*^*th*^ order statistic, i.e *X*_(1)_ < *X*_(2)_ <…< *X*_(*n*)_. If *p*_*i*_ denotes some estimate of *F*(*x*_(*i*)_) then the percentiles estimates, ${\hat{\alpha }}_{PC}$, ${\hat{\beta }}_{PC}$, ${\hat{\gamma }}_{PC}$ of *α*, *β* and *γ* can be obtained by minimizing

$$\begin{array}{c} PC(\zeta )=\sum_{i=1}^{n}{\left[x_{\left(i\right)}-\gamma^{\frac{1}{\beta }}{\left\{{\left(p_{i}\right)}^{-\frac{\gamma }{\alpha }}-1\right\}}^{\frac{-1}{\beta }}\right]}^{2};i=1,2,\ldots ,n. \end{array}$$ (27)

Several estimators of *p*_*i*_ can be used. In this paper, we consider *p*_*i*_ = $\frac{i}{n+1}$.

### Maximum product of spacings estimates (MPSE)

[60, 61] introduced the maximum product of spacings (MPS) method as an alternative to MLE for the estimation of parameters of continuous uni-variate distributions. [62] independently developed the same method as an approximation for the Kullback-Leibler measure of information. This method is constructed on a clue that differences (spacings) between the values of the cdf at consecutive data points should be identically distributed. [60] proved that this method is as efficient as the MLEs and consistent under more general conditions. The geometric mean of the differences is given as

$$\begin{array}{c} G.M=\sqrt[{n+1}]{\prod_{i=1}^{n+1}D_{i}}, \end{array}$$

where, the difference *D*_*i*_ is defined as

$$\begin{array}{c} D_{i}=\int_{x_{\left(i-1\right)}}^{x_{\left(i\right)}}f(x)dx;i=1,2,\ldots ,n+1. \end{array}$$ (28)

The maximum product spacing (MPS) estimates, ${\hat{\alpha }}_{MPS}$, ${\hat{\beta }}_{MPS}$, ${\hat{\gamma }}_{MPS}$, of *α*, *β* and *γ* are obtained by maximizing the geometric mean of the differences. Incorporating cdf of hcMB−III distribution in Eq (28) and taking logarithm of the above expression, we have

$$\begin{array}{c} MPS(\zeta )=\frac{1}{n+1}\sum_{i=1}^{n+1}\;\text{log}[F(x_{\left(i\right)})-F(x_{\left(i-1\right)})],i=1,2,\ldots ,n+1. \end{array}$$ (29)

where, *F*(*x*_(0)_) = 0 and *F*(*x*_(*n*+1)_) = 1. By maximizing *MPS*(*ζ*), the MPSEs ${\hat{\alpha }}_{MPS}$, ${\hat{\beta }}_{MPS}$, ${\hat{\gamma }}_{MPS}$ are attained.

### Minimum distances estimators

This section presents three estimation methods for *α*, *β* and *γ* based on the minimization of the goodness−of−fit statistics. This class of statistics is based on the difference between the estimate of the cumulative distribution function and the empirical distribution function [63].

#### Cramér-von-Mises estimates (CVME)

To motivate our choice of Cramér-von-Mises type minimum distance estimators, [64] provided empirical evidence that the bias of the estimator is smaller than the other minimum distance estimators. Thus, the Cramér-von-Mises estimates, ${\hat{\alpha }}_{CVM}$, ${\hat{\beta }}_{CVM}$, ${\hat{\gamma }}_{CVM}$, of *α*, *β* and *γ* are obtained by minimizing

$$\begin{array}{c} CVM(\zeta )=\frac{1}{12n}+\sum_{i=1}^{n}{\left[F(x_{\left(i\right)})-\frac{2i-1}{2n}\right]}^{2}. \end{array}$$ (30)

#### Anderson-Darling estimates(ADE)

The Anderson-Darling(AD) test was developed by [65] as an alternative to other statistical tests for detecting sample distributions departure from normality. It is interesting to note that the Anderson-Darling test converges so quickly towards the asymptote [65]. The Anderson-Darling estimates ${\hat{\alpha }}_{AD}$, ${\hat{\beta }}_{AD}$, ${\hat{\gamma }}_{AD}$, of *α*, *β* and *γ* are obtained by minimizing the following function

$$\begin{array}{c} AD(\zeta )=-n-\sum_{i=1}^{n}\frac{2i-1}{n}[\text{log}F(x_{\left(i\right)})+\text{log}\{1-F(x_{\left(n+1-i\right)})\}]. \end{array}$$ (31)

#### Right-tail Anderson-Darling estimates (RTAD)

The Right-tail Anderson-Darling (RTAD) estimates of ${\hat{\alpha }}_{RTAD}$, ${\hat{\beta }}_{RTAD}$, ${\hat{\gamma }}_{RTAD}$, of *α*, *β* and *γ* are obtained by minimizing the following function

$$\begin{array}{c} RTAD(\zeta )=\frac{n}{2}-2\sum_{i=1}^{n}F(x_{\left(i\right)})-\frac{1}{n}\sum_{i=1}^{n}\{(2n-1)log\bar{F}(x_{\left(n+1-i\right)})\} \end{array}$$ (32)

## Simulation study for the comparison of different estimation methods

**Fig 5:**
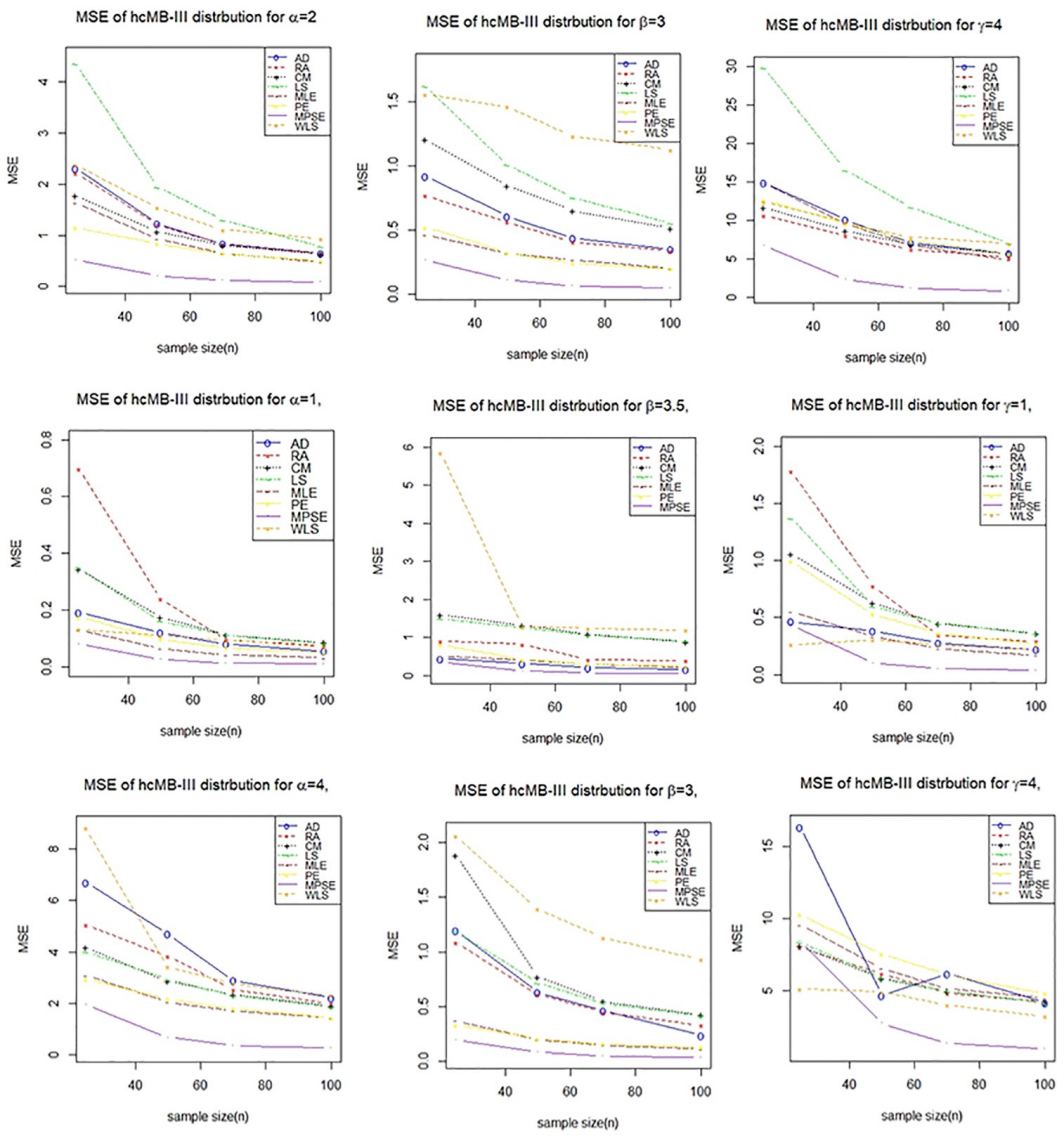
MSE for different sample sizes and estimation methods for different parameters values of (*α*, *β*, *γ*).

This section presents simulation studies by using the hcMB−III distribution to assess the performance of the above estimators discussed in the previous section and obtained numerical and graphical results. We generate *N* = 10, 000 samples of the size *n* = (25, 50, 75, 100) from hcMB−III distribution with parameter settings (*α*, *β*, *γ*) = {(2, 3, 4), (1, 3.5, 1), (4, 3, 4)}. The random numbers generation is obtained by its quantile function. In this simulation study, we calculate the empirical mean, bias and mean square errors (MSEs) of all estimators to compare in the terms of their biases and MSEs with varying sample size. It is noticed that 10,000 iterations are sufficiently large to have stable results. The empirical bias and MSE are calculated by (*for t* = *α*, *β*, *γ*)

$$\begin{array}{c} {\hat{Bias}}_{t}=\frac{1}{N}\sum_{i=1}^{N}({\hat{t}}_{i}-t) \end{array}$$

and

$${\hat{MSE}}_{t}=\frac{1}{N}\sum_{i=1}^{N}{\left({\hat{t}}_{i}-t\right)}^{2}$$

respectively. All results related to estimation were obtained by using software Mathematica 12.0. The results of simulations are shown in Fig 5 in which comparison of MSE on the basis of different sample size and estimation is presented for different values of (*α*, *β*, *γ*). It is also worth noting that MSE is reducing for large sample size. MPSE has smallest MSE for all parameters setting as compared to other estimation methods used for comparison. LSE and RTADE also have high MSE for all parameters. PCE has high MSE for *β*=3 and 3.5. Most suitable methods are MLE, ADE, PCE and MPSE for hcMB−III distribution.

Simulation results for hcMB−III distribution in terms of mean, bias, mean square error and standard error is given to compare performance of different estimation methods for different parameter settings S2–S4 Appendices.

## Application

**Fig 6:**
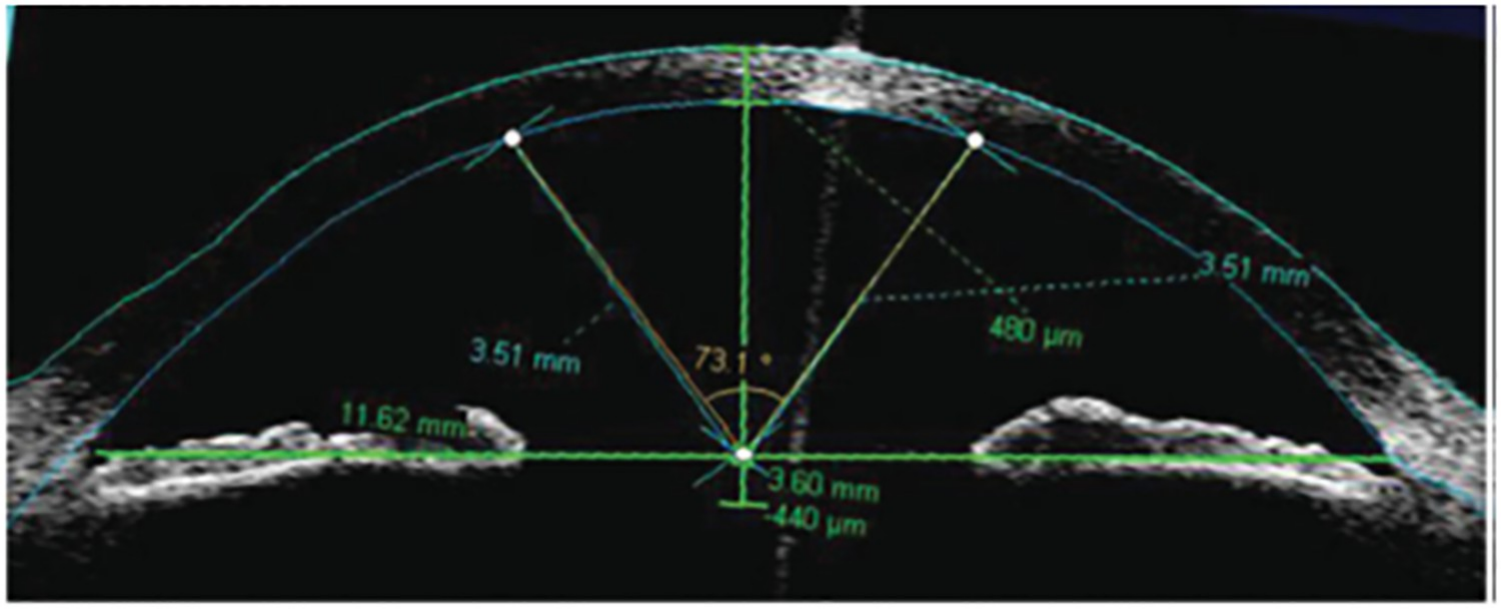
Images of posterior corneal curvature measurement.

**Fig 7:**
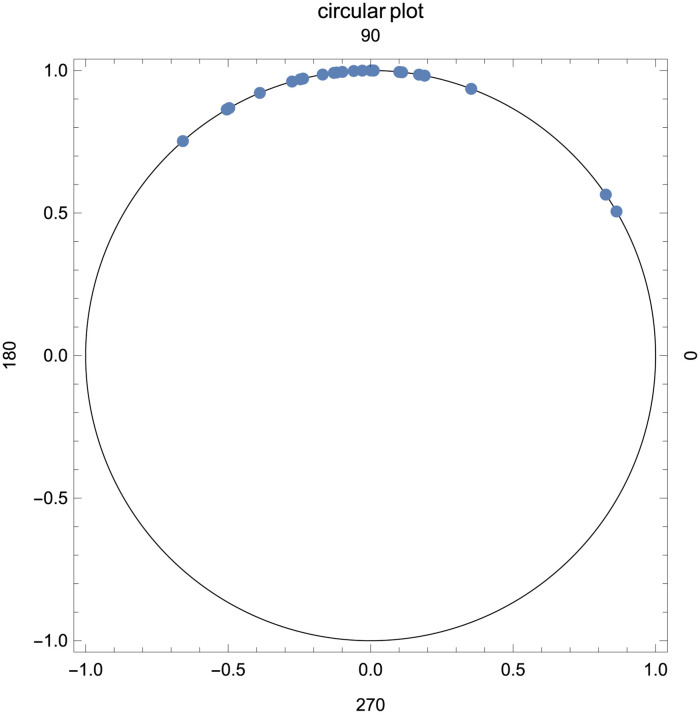
Circular plot of eye data.

In this section, we consider the data obtained from the images of the posterior segment of the eyes of 23 patients. This data set has also been analyzed by [22, 27, 28, 66]. The data is also available in S1 Data. The half circular variable of our interest is the angle which measures the posterior corneal curvature defined below. Fig 6 presents an image of the posterior segment, where O is the intersection of the geometrical axis of the eye (horizontal line) with the line made between the nasal and temporal scleral spurs (vertical line). The circular plot is given in Fig 7, where it is obvious that the angles are concentrated in the first and second quadrant with range 1.76, which confirms that the data is a random sample from a half circular distribution, where *θ* ∈ [0, *π*).

We compute MLEs and their standard errors (S.Es) in parenthesis for hcMB−III distribution, half-circular gamma distribution, half-circular Burr-XII distribution and sub models of hcMB −III distribution are given in Table 2. For the selection of the best fit model, we compute the estimate of likelihood ratio statistic $-2\;\ell (\hat{\Theta })$, AIC, CAIC, BIC, Anderson-Darling (A*), Cràmer-von-Mises (A*) and Kolmogorov- Smirnove (K-S) statistic along with its p-value for all competing models. All models are evaluated at MLE by using **FitdistrPlus, AdequacyModel, Pracma** packages in R and the results are presented in Table 3. Among all other competitive models, it is noted that the hcMB−III distribution has the smallest values of $-2\;\ell (\hat{\Theta })$, AIC, BIC and CAIC. It is also noted that hcMB−III distribution has lowest value of (A*), (W*) and K-S (p-value) which indicates that it best fits the given eye data set than other half-circular distribution being used for comparison.

**Table 2:** ML estimates and S.E (in parentheses) for eye data.

Distribution	ML estimates and S.E (in parenthesis)		
**hc−MBIII** (*α*, *β*, *γ*)	2.698357 (2.199659)	6.649816 (2.002855)	6.286918 (7.688339)
**hc−BurrIII** (*α*, *β*)	1.000473 (0.2294769)	4.286727 (0.8573875)	——–
**hc−GIW** (*α*, *β*, *γ*)	0.823392 (52.211295)	1.702156 (0.2366994)	0.910867 (98.313168)
**hc−LL**(*α*, *β*)	1.064250 (0.084368)	4.384939 (0.80058078)	——–
**hc−Gamma**(*α*, *β*)	5.7177171 (1.638393)	0.193358 (0.057909)	——-
**hc−Burr XII1**(c, k)	4.379365 (0.890822)	0.947472 (0.220552)	——–

**Table 3:** $-2\;l(\hat{\Theta })$ , AIC, CAIC, BIC, A*, w*, K-S (p-values) for eye data.

Model	$-2\;l(\hat{\Theta })$	AIC	CAIC	BIC	A*	W*	K-S (p-value)
**hc-MBIII**	19.23826	25.23826	26.50142	28.64474	0.4451791	0.067782	0.127358 (0.849687)
**hc-Burr III**	22.74478	26.74478	27.34478	29.01577	0.8152383	0.11913	0.183918(0.417999)
**hc-GIW**	37.54608	43.54607	44.80923	46.95255	2.42950	0.41253	0.27321(0.064537)
**hc-Log logistic**	22.14424	26.14425	26.74425	28.41524	0.74076	0.10753	0.116541(0.913587)
**hc-Gamma**	22.17462	26.17461	26.77461	28.4456	0.8166877	0.127228	0.169892(0.520301)
**hc-Burr-XII**	22.69012	26.69013	27.29013	28.96112	0.818624	0.119508	0.16551(0.55440)

**Fig 8:**
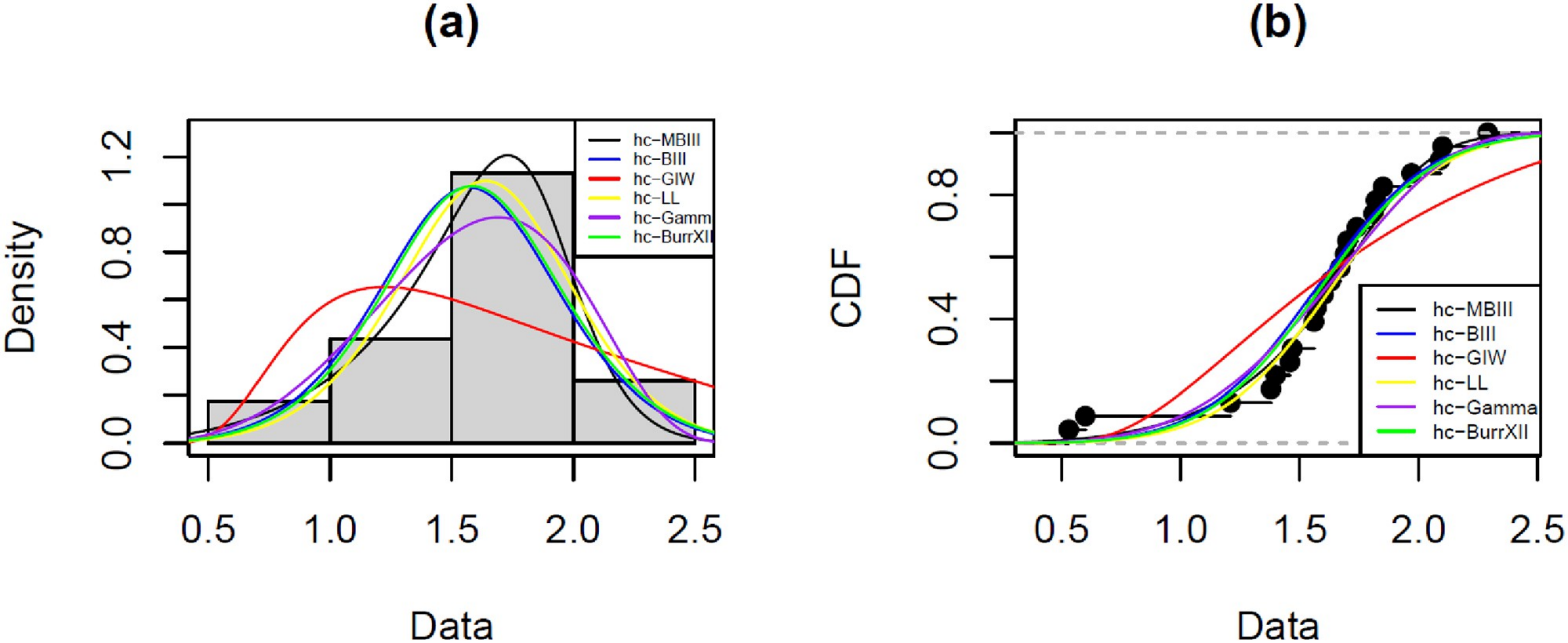
The fitted pdf of hcMB−III model and other models and cdf of hcMB−III model on eye data. A:Fitted densities of hcMB−III distribution and other models on eye data. B:Estimated cdfs of hcMB−III distribution and other models on eye data.

**Fig 9:**
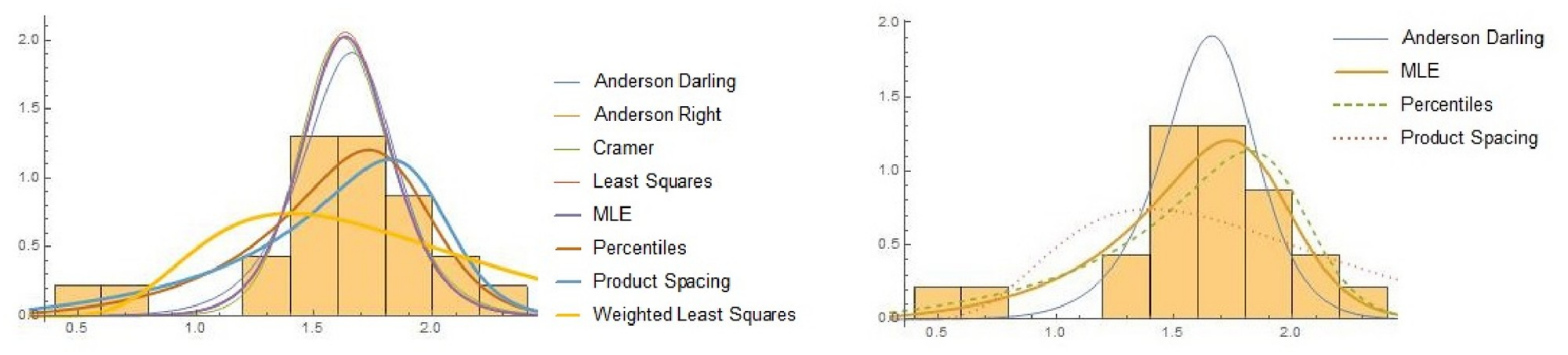
A:Fitting of hcMB−III distribution with different Estimation Methods. B: Behavior of selected estimation methods for hcMB−III distribution.

Fig 8 shows the fitted models vs hcMB−III distribution along with cdfs of all competing models. We fitted the hcMB−III distribution using the eight estimation methods. The parameter estimates for eye data set are reported in Table 4. Descriptive statistics for different estimated values of hcMB−III distribution (*α*, *β*, *γ*) for all estimation methods are given in Table 5 and we observe that CVME has smallest variance among all others while MPSE has smallest value of skewness. A graphical presentation of fitting above mentioned estimation methods is shown in Fig 9. It is evident from the tables and figures that the hcMB−III distribution provides better fit as compared to other existing models considered here.

**Table 4:** Parameters estimation through different estimation methods for eye data.

	*α*	*β*	*γ*
**ADE**	2.044690	8.364370	2.703020
**RTADE**	1.353820	7.036390	0.790281
**CVME**	1.595240	7.927040	1.407220
**LSE**	1.560780	7.734630	1.342880
**MLE**	2.697290	6.649040	6.283080
**PCE**	5.726210	7.437220	19.17870
**MPSE**	2.507843	5.818691	5.982744
**WLSE**	1.632090	7.915530	1.513830

**Table 5:** Descriptive statistics for different parameters estimates methods.

	ADE	RTADE	CVME	LSE	MLE	PCE	MPSE	WLSE
**Mean Direction** (*μ*)	-1.5111	-1.49429	-1.5042	-1.5037	-1.5491	-1.5429	-1.4953	-1.5048
**Variance** (*υ*)	0.027	0.02248	0.02218	0.0229	0.0697	0.0878	0.1266	0.023
**Trigonometric Moment** (*α*_1_)	-0.058	-0.07474	-0.065	-0.0655	-0.0201	-0.0255	-0.0659	-0.0644
**Trigonometric Moment** (*α*_2_)	-0.8906	-0.90337	-0.9067	-0.9041	-0.7513	-0.6947	-0.57	-0.904
**Trigonometric Moment** (*β*_1_)	0.97127	0.97465	0.97565	0.9749	0.93008	0.9119	0.8709	0.9749
**Trigonometric Moment** (*β*_2_)	-0.1103	-0.13641	-0.121	-0.1213	-0.0602	-0.087	-0.0453	-0.1199
**Resultant length** (*ρ*)	0.973	0.97752	0.97782	0.9771	0.9303	0.9122	0.8734	0.9771
**Skewness** (*γ*_1_)	202.278	270.98	276.913	263.7	40.9367	26.855	12.657	262.3
**Kurtosis** (*γ*_2_)	1.50596	1.09873	1.20532	1.1657	0.87288	0.784	-0.7168	1.2121

**Fig 10:**
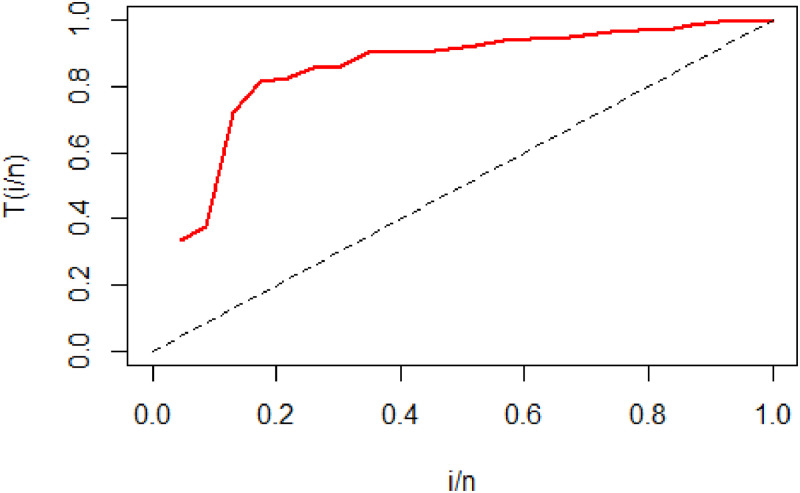
TTT plot for eye data set.

**Fig 11:**
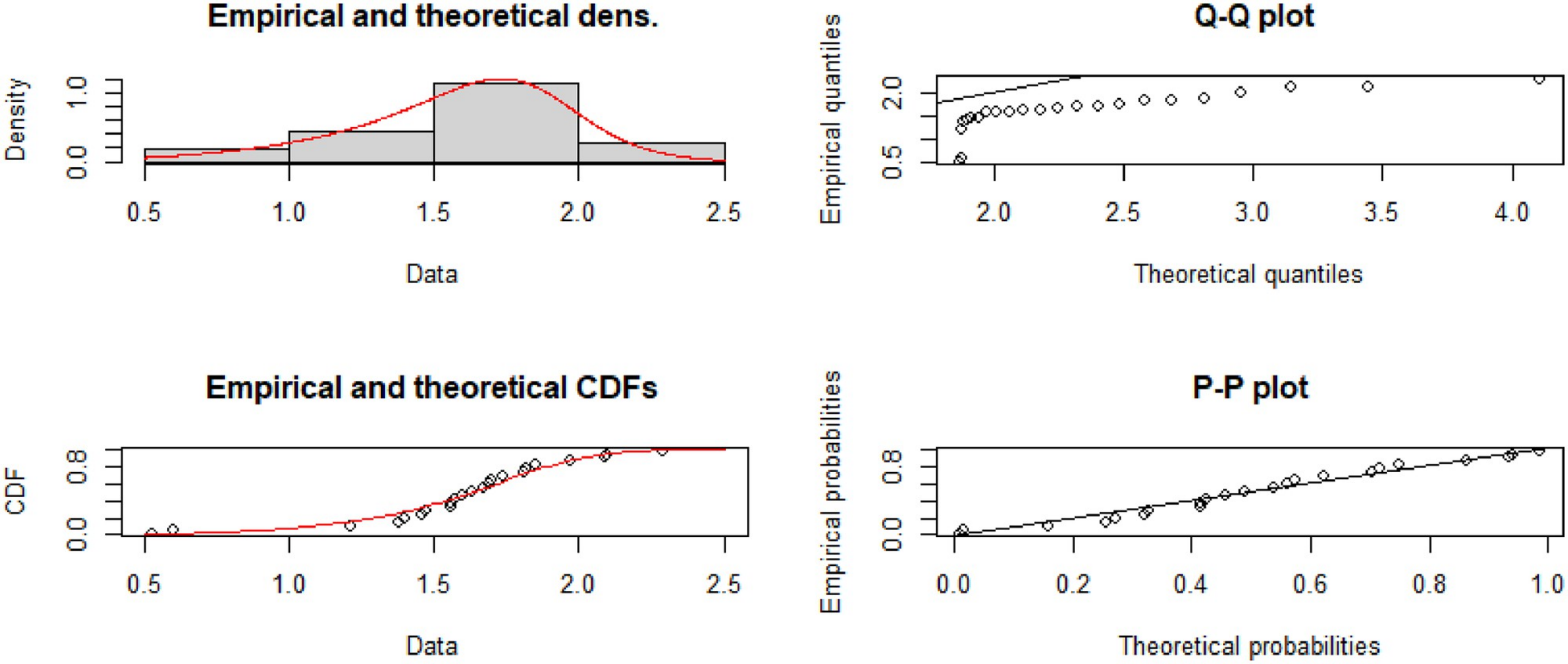
Fitted pdf, cdf, qq and pp plots of the hcMB−III distribution for eye data.

To check the shape of the hazard function of the data set, we have used TTT (Total time on test) plot proposed by [67] in Fig 10 indicates that the data set has increasing hazard rate. Also see [68] for more details about parameter estimation and TTT plot. We can perceive that the hcMB−III distribution is best fitted to empirical data Fig 11

## Conclusions

Circular data is used in measuring observations arising in the different fields of science. Due to the wide range of applications for half-circular data, it is still worth exploring it further. In this paper, a new half circular distribution is proposed based on an inverse stereographic projection technique applied on the distribution of MB−III. Various properties of suggested distribution are derived. The parameter estimates are obtained by employing the eight estimation methods. The estimation methods were compared on the basis of their Means, Average biases, MSEs and Standard errors for different parameters settings. We perform simulation studies on the basis of the graphical as well as numerical results to see the performances of the estimates of hcMB− III distribution. Different properties such as mean direction, variance, trigonometric moments, resultant length, skewness and kurtosis are calculated for all eight estimation methods. The suggested model best fits the eye data of 23 patients’ posterior corneal curvature when compared to existing semi-circular models and sub models of hcMB− III distribution. The potentiality of hcMB− III distribution illustrates that it is flexible, competitive and parsimonious. Thus, it should be included in the distribution theory to facilitate researchers and practitioners dealing with angular data. Further, as perspective of future projects, we may study some rigorous issues (i)Characterization of proposed model can be done by using different methods (ii)Outliers detection. (iii)ℓ-axial half circular Modified Burr III can be studied. (iv)Sub models of half circular distribution can be explored in detail. (v)Bayesian analysis can be performed to study complexity of the proposed model. (vi)Bi-variate case of hcMB−III distribution may also be studied.

## Supporting information

S1 Appendix Table 6: Characteristics of hcMB−III distribution for different parameter values. (TIF)

S2 Appendix Table 7: Mean, average bias, standard error and MSE for different estimation methods for hcMB−III distribution (*α*, *β*,*γ*) = (2,3,4). (TIF)

S3 Appendix Table 8: Mean, average bias, standard error and MSE for different estimation methods for hcMB−III distribution (*α*, *β*,*γ*) = (1, 3.5, 1). (TIF)

S4 Appendix Table 9: Mean, average bias, standard error and MSE for different estimation methods for hcMB−III distribution (*α*, *β*, *γ*) = (4,3,4). (TIF)

S1 Data Excel file for eye data set used in data analysis. (XLSX)

## References

[1] Jones TA, James WR. Analysis of bimodal orientation data. Journal of the International Association for Mathematical Geology. 1969;1(2):129–135. doi: 10.1007/BF02048557

[2] Rao JS, Sengupta S. Mathematical techniques for paleocurrent analysis: treatment of directional data. Journal of the International Association for Mathematical Geology. 1972;4(3):235–248. doi: 10.1007/BF02311720

[3] Breckling J. The analysis of directional time series: applications to wind speed and direction. vol. 61. Springer Science & Business Media; 2012.

[4] Brunsdon C, Corcoran J. Using circular statistics to analyse time patterns in crime incidence. Computers, Environment and Urban Systems. 2006;30(3):300–319. doi: 10.1016/j.compenvurbsys.2005.11.001

[5] Abuzaid AH. Analysis of Mother’s Day celebration via circular statistics. The Philippine Statistician. 2012;61(2):39–52.

[6] Mardia KV. Statistics of directional data. Journal of the Royal Statistical Society: Series B (Methodological). 1975;37(3):349–371.

[7] Fisher NI. Statistical analysis of circular data. cambridge university press; 1995.

[8] Lund U. Least circular distance regression for directional data. Journal of Applied Statistics. 1999;26(6):723–733. doi: 10.1080/02664769922160

[9] Jammalamadaka SR, Sengupta A. Topics in circular statistics. vol. 5. world scientific; 2001.

[10] Minh DL, Farnum NR. Using bilinear transformations to induce probability distributions. Communications in Statistics-Theory and Methods. 2003;32(1):1–9.

[11] Jones MC, Pewsey A. A family of symmetric distributions on the circle. Journal of the American Statistical Association. 2005;100(472):1422–1428. doi: 10.1198/016214505000000286

[12] Jammalamadaka SR, Kozubowski TJ. A general approach for obtaining wrapped circular distributions via mixtures. Sankhya A. 2017;79(1):133–157. doi: 10.1007/s13171-017-0096-4

[13] Joshi S, Jose KK. Wrapped lindley distribution. Communications in Statistics-Theory and Methods. 2018;47(5):1013–1021. doi: 10.1080/03610926.2017.1280168

[14] Yedlapalli P, Girija S, Rao A. On construction of stereographic semicircular models. Journal of Applied Probability. 2013;8(1):75–90.

[15] Ugai S, Kato K, Nishijima M, Kan T. Characteristics of raindrop size and raindrop shape. In: Union Radio Scientifique Internationale, Open Symposium, La Baule, Loire-Atlantique, France; 1977. p. 225–230.

[16] Guardiola JH. The semicircular normal distribution. Baylor University; 2004.

[17] Ahn BJ, Kim HM. A New Family of Semicircular Models: The Semicircular Laplace Distributions. Communications for Statistical Applications and Methods. 2008;15(5):775–781. doi: 10.5351/CKSS.2008.15.5.775

[18] Kim HM. New Family of the Exponential Distributions for Modeling Skewed Semicircular Data; 2009.

[19] Kim HM. A Projected Exponential Family for Modeling Semicircular Data. Korean Journal of Applied Statistics. 2010;23(6):1125–1145. doi: 10.5351/KJAS.2010.23.6.1125

[20] Girija SVS, Rao AVD, Yedlapalli P. New Circular model induced by Inverse Stereographic projection on Double Exponential Model—Application to Birds Migration Data. Journal of Applied Mathematics, Statistics and Informatics. 2014;10(1):5–17. doi: 10.2478/jamsi-2014-0001

[21] Pramesti G. The Stereographic Semicircular Chi Square Models. Far East Journal of Theoretical Statistics. 2015;51(3):119. doi: 10.17654/FJTSNov2015_119_128

[22] Rambli A, Mohamed IB, Shimizu K, Khalidin N. Outlier detection in a new half-circular distribution. In: AIP Conference Proceedings. vol. 1682. AIP Publishing LLC; 2015. p. 050018.

[23] Rao AVD, Girija SVS, Phani Y. Stereographic Logistic Model—Application to Noisy Scrub Birds Data. Chilean Journal of Statistics. 2016;7(2):69–79.

[24] Pramesti G, Jin Y. Exponential Circular Distribution Motivated by Inverse Stereographic Projection. International journal of applied mathematics and statistics. 2016;54:114–122.

[25] Yedlapalli P, Girija SVS, Rao AVD. ON STEREOGRAPHIC CIRCULAR WEIBULL DISTRIBUTION. Journal of New Theory. 2016;14(1):1–9.

[26] Subrahmanyam PS, Rao AVD, Girija SVS. ON STEREOGRAPHIC SEMICIRCULAR EXPONENTIATED INVERTED. International Journal of Advanced Research and Review. 2017;2(5):46–56.

[27] Abuzaid AH. A half circular distribution for modeling the posterior corneal curvature. Communications in Statistics-Theory and Methods. 2018;47(13):3118–3124. doi: 10.1080/03610926.2017.1348521

[28] Rambli A, Mohamed I, Shimizu K, Ramli NM. A half-circular distribution on a circle. Sains Malaysiana. 2019;48(4):887–892. doi: 10.17576/jsm-2019-4804-21

[29] Phani Yedlapalli, Girija V S, Rao V D, Akkayajhula, Sastry KLN. On Stereographic Semicircular Quasi Lindley Distribution. JOURNAL OF NEW RESULTS IN SCIENCE. 2019;8(2014):6–13.

[30] Yedlapalli P, Girija SVS, Dattatreya Rao AV, Sastry KLN. A new family of semicircular and circular arc tan-exponential type distributions. Thai Journal of Mathematics. 2020;18(2):775–781.

[31] Burr IW. Cumulative frequency functions. The Annals of mathematical statistics. 1942;13(2):215–232. doi: 10.1214/aoms/1177731607

[32] Momanyi RO, Ottieno J. Generating Distribution Functions Based on Burr Differential Equation. Journal of Advances in Mathematics and Computer Science. 2020; p. 55–64. doi: 10.9734/jamcs/2020/v35i830313

[33] Korkmaz MÇ, Altun E, Yousof HM, Afify AZ, Nadarajah S. The Burr X Pareto Distribution: Properties, Applications and VaR Estimation. Journal of Risk and Financial Management. 2018;11(1):1. doi: 10.3390/jrfm11010001

[34] Nasir MA, Korkmaz MC, Jamal F, Yousof HM. On A New Weibull Burr XII Distribution for Lifetime Data. Sohag Journal of Mathematics. 2018;5(2):47–56. doi: 10.18576/sjm/050202

[35] Bhatti FA, Hamedani GG, Korkmaz MC, Cordeiro GM, Yousof HM, Ahmad M. On Burr III Marshal Olkin family: development, properties, characterizations and applications. Journal of Statistical Distributions and Applications. 2019;6(1). doi: 10.1186/s40488-019-0101-7

[36] Bhatti FA, Ali A, Hamedani GG, Korkmaz M, Ahmad M. The unit generalized log burr xii distribution: Properties and application. AIMS Mathematics. 2021;6(9):10222–10252. doi: 10.3934/math.2021592

[37] Korkmaz M, Chesneau C. On the unit Burr-XII distribution with the quantile regression modeling and applications. Computational and Applied Mathematics. 2021;40(1):1–26. doi: 10.1007/s40314-021-01418-5

[38] Bhatti FA, Hamedani GG, Korkmaz M, Sheng W, Ali A. On the Burr XII-moment exponential distribution. PLoS ONE. 2021;16(2 February):1–21. doi: 10.1371/journal.pone.0246935 33617564 PMC7899380

[39] Mielke PW Jr. Another family of distributions for describing and analyzing precipitation data. Journal of Applied Meteorology and Climatology. 1973;12(2):275–280. doi: 10.1175/1520-0450(1973)012<0275:AFODFD>2.0.CO;2

[40] Dagum C. New model of personal income-distribution-specification and estimation. Economie appliquée. 1977;30(3):413–437.

[41] Abdel-Ghaly AA, Al-Dayian GR, Al-Kashkari FH. The use of burr type XII distribution on software reliability growth modelling. Microelectronics Reliability. 1997;37(2):305–313. doi: 10.1016/0026-2714(95)00124-7

[42] Chernobai GB, Chesalov YA, Burgina EB, Drebushchak TN, Boldyreva EV. Temperature effects on the IR spectra of crystalliine amino acids, dipeptides, and polyamino acids. I. Glycine. Journal of Structural Chemistry. 2007;48(2):332–339. doi: 10.1007/s10947-007-0050-8

[43] Gove JH, Ducey MJ, Leak WB, Zhang L. Rotated sigmoid structures in managed uneven-aged northern hardwood stands: a look at the Burr Type III distribution. Forestry. 2008;81(2):161–176. doi: 10.1093/forestry/cpm025

[44] Bhatti FA, Ahmad M. On Burr III—Pareto Distribution: Development, Properties, Characterizations and Applications. Pakistan Journal of Statistics and Operation Research. 2019;15(2):371–395.

[45] Handique L, Usman R, Chakraborty S. New Extended Burr-III Distribution: Its Properties and Applications. Thailand Statistician. 2020;18(3):267–280.

[46] Ali A, Hasnain SA, Ahmad M. Pak. J. Statist. 2015 Vol. 31 (6), 697-708 MODIFIED BURR III DISTRIBUTION, PROPERTIES AND APPLICATIONS. Pak J Statist. 2015;31(6):697–708.

[47] Ali A, Ahmad M. Journal of ISOSS 2015 Vol. 1 (2), 119-130 THE TRANSMUTED MODIFIED BURR III DISTRIBUTION. Journal of ISOSS. 2015;1(2):119–130.

[48] Bhatti FA, Hamedani G, Ali A, Ahmad M. Some characterizations of transmuted modified Burr III distribution. Asian Journal of Probability and Statistics. 2018; p. 1–9. doi: 10.9734/ajpas/2018/v1i124498

[49] Arifa S, Yab MZ, Ali A. The modified Burr III G family of distributions. Journal of Data Science. 2017;15(1):41–60. doi: 10.6339/JDS.201701_15(1).0003

[50] Ali A, Dey S, Rehman HU, Ali Z. On Bayesian reliability estimation of a 1-out-of-k load sharing system model of modified Burr-III distribution. International Journal of System Assurance Engineering and Management. 2019;10(5):1052–1081.

[51] Mukhtar S, Ali A, Alya AM. Mc-Donald modified Burr-III distribution: properties and applications. Journal of Taibah University for Science. 2019;13(1):184–192. doi: 10.1080/16583655.2018.1553501

[52] Bhatti FA, Hamedani GG, Sheng W, Ahmad M. Cubic rank transmuted modified burr III pareto distribution: Development, properties, characterizations and applications. International Journal of Statistics and Probability. 2019;.

[53] ul Haq MA, Hashmi S, Aidi K, Ramos PL, Louzada F. Unit modified Burr-III distribution: Estimation, characterizations and validation test. Annals of Data Science. 2020; p. 1–26.

[54] Bhatti FA, Hamedani GG, Korkmaz MC, Ahmad M. On the new modified Dagum distribution: Properties and applications. Journal of Statistics and Management Systems. 2020;23(8):1513–1542. doi: 10.1080/09720510.2020.1745390

[55] ul Haq MA, Afify AZ, Al-Mofleh H, Usman RM, Alqawba M, Sarg AM. The Extended Marshall-Olkin Burr III Distribution: Properties and Applications. Pakistan Journal of Statistics and Operation Research. 2021; p. 1–14. doi: 10.18187/pjsor.v17i1.3649

[56] Mardia K. Directional Statistics by KV Mardia & PE Jupp Wiley, Chichester, 2000.; 2000.

[57] Swain JJ, Venkatraman S, Wilson JR. Least-squares estimation of distribution functions in Johnson’s translation system. Journal of Statistical Computation and Simulation. 1988;29(4):271–297. doi: 10.1080/00949658808811068

[58] Kao JHK. Computer methods for estimating Weibull parameters in reliability studies. IRE Transactions on Reliability and Quality Control. 1958; p. 15–22. doi: 10.1109/IRE-PGRQC.1958.5007164

[59] Kao JHK. A graphical estimation of mixed Weibull parameters in life-testing of electron tubes. Technometrics. 1959;1(4):389–407. doi: 10.1080/00401706.1959.10489870

[60] Cheng RCH, Amin NAK. Maximum product of spacings estimation with applications to the lognormal distribution. Math report. 1979;791.

[61] Cheng RCH, Amin NAK. Estimating parameters in continuous univariate distributions with a shifted origin. Journal of the Royal Statistical Society: Series B (Methodological). 1983;45(3):394–403.

[62] Ranneby B. The maximum spacing method. An estimation method related to the maximum likelihood method. Scandinavian Journal of Statistics. 1984; p. 93–112.

[63] Luceño A. Fitting the generalized Pareto distribution to data using maximum goodness-of-fit estimators. Computational Statistics & Data Analysis. 2006;51(2):904–917. doi: 10.1016/j.csda.2005.09.011

[64] Macdonald PDM. Comments and queries comment on “an estimation procedure for mixtures of distributions” by choi and bulgren. Journal of the Royal Statistical Society: Series B (Methodological). 1971;33(2):326–329.

[65] Anderson TW, Darling DA. A test of goodness of fit. Journal of the American statistical association. 1954;49(268):765–769. doi: 10.1080/01621459.1954.10501232

[66] Abuzaid AH. Identifying density-based local outliers in medical multivariate circular data. Statistics in Medicine. 2020;39(21):2793–2798. doi: 10.1002/sim.8576 32430937

[67] Aarset MV. How to Identify a Bathtub Hazard Rate. IEEE Transactions on Reliability. 1987;R-36(1):106–108. doi: 10.1109/TR.1987.5222310

[68] Marinho PRD, Silva RB, Bourguignon M, Cordeiro GM, Nadarajah S. AdequacyModel: An R package for probability distributions and general purpose optimization. PLoS ONE. 2019;14(8):1–30. doi: 10.1371/journal.pone.0221487 PMC6710032 31450236

