# Supplementary figures and images for: Half circular modified burr−III distribution, application with different estimation methods
Source: PLoS One. 2022 May 17;17(5):e0261901. doi: 10.1371/journal.pone.0261901 (PMC9113593; doi:10.1371/journal.pone.0261901)

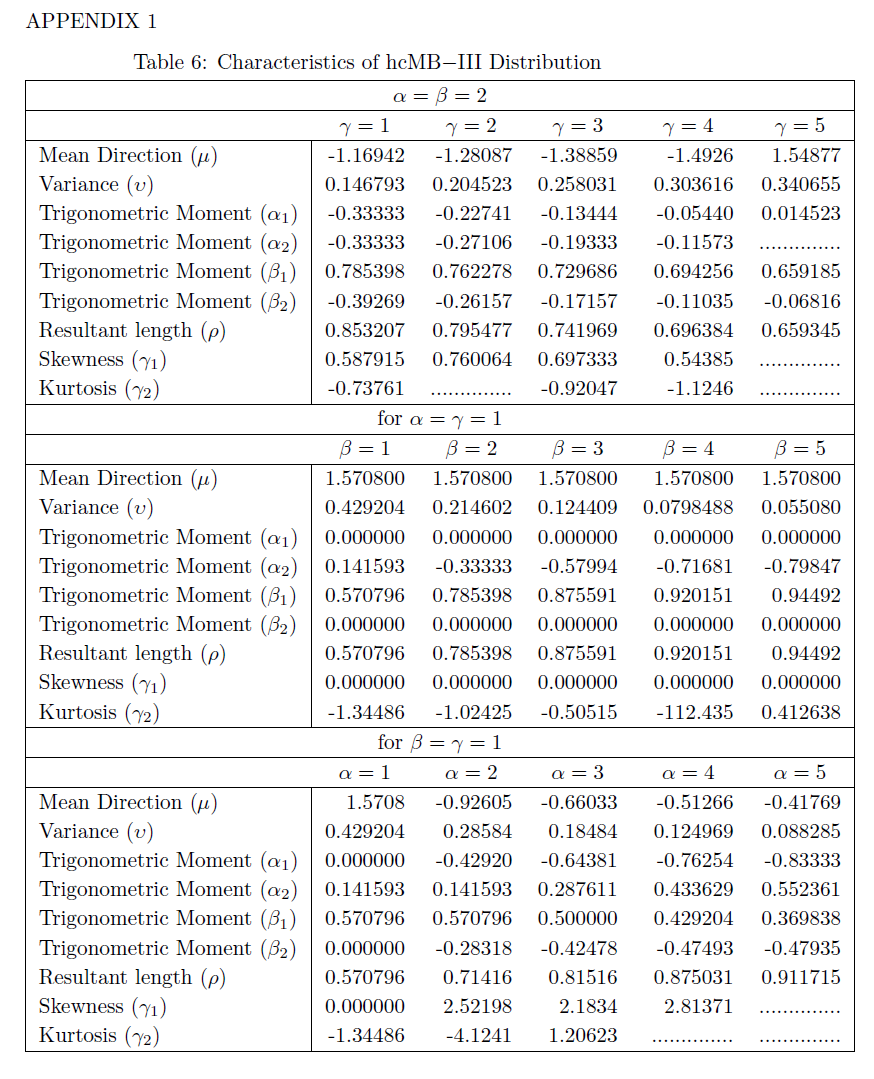

Supplement: S1 Appendix — (TIF) [file pone.0261901.s001.tif]

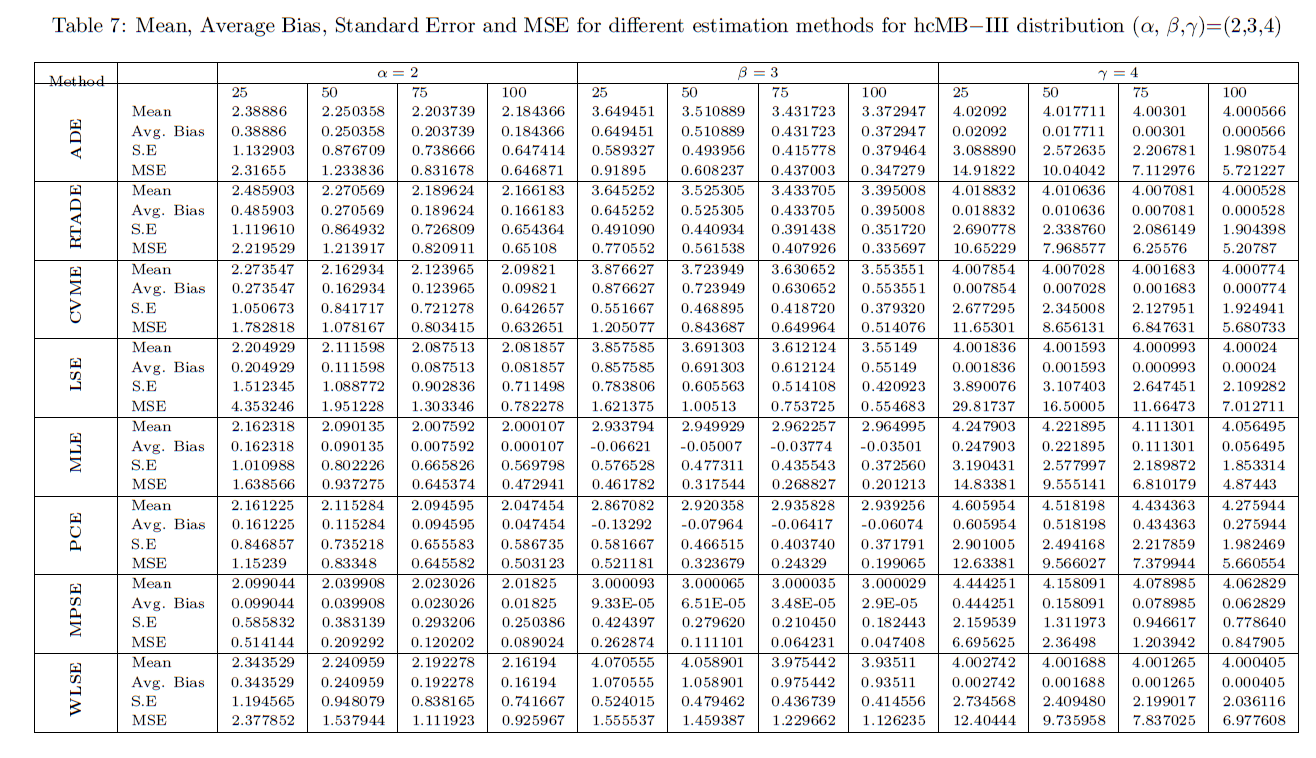

Supplement: S2 Appendix — (TIF) [file pone.0261901.s002.tif]

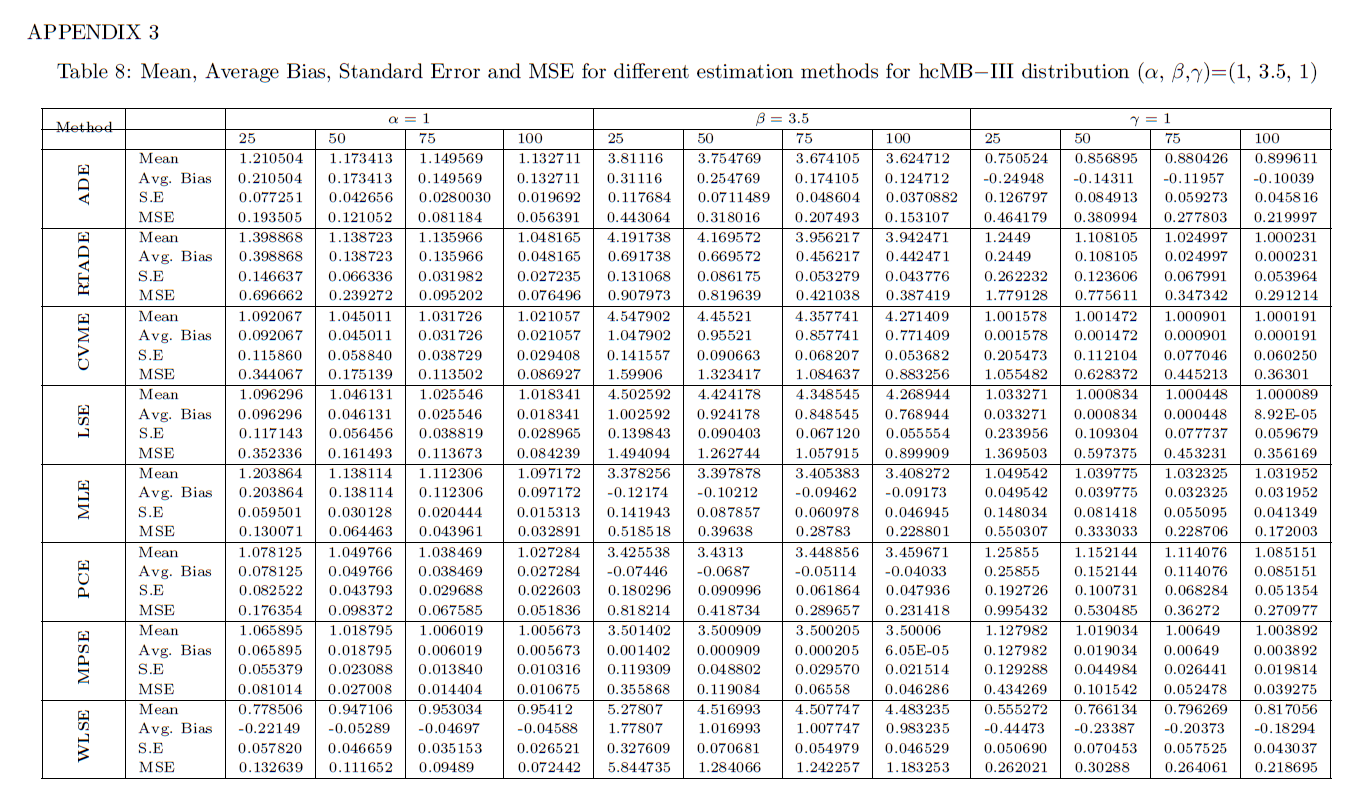

Supplement: S3 Appendix — (TIF) [file pone.0261901.s003.tif]

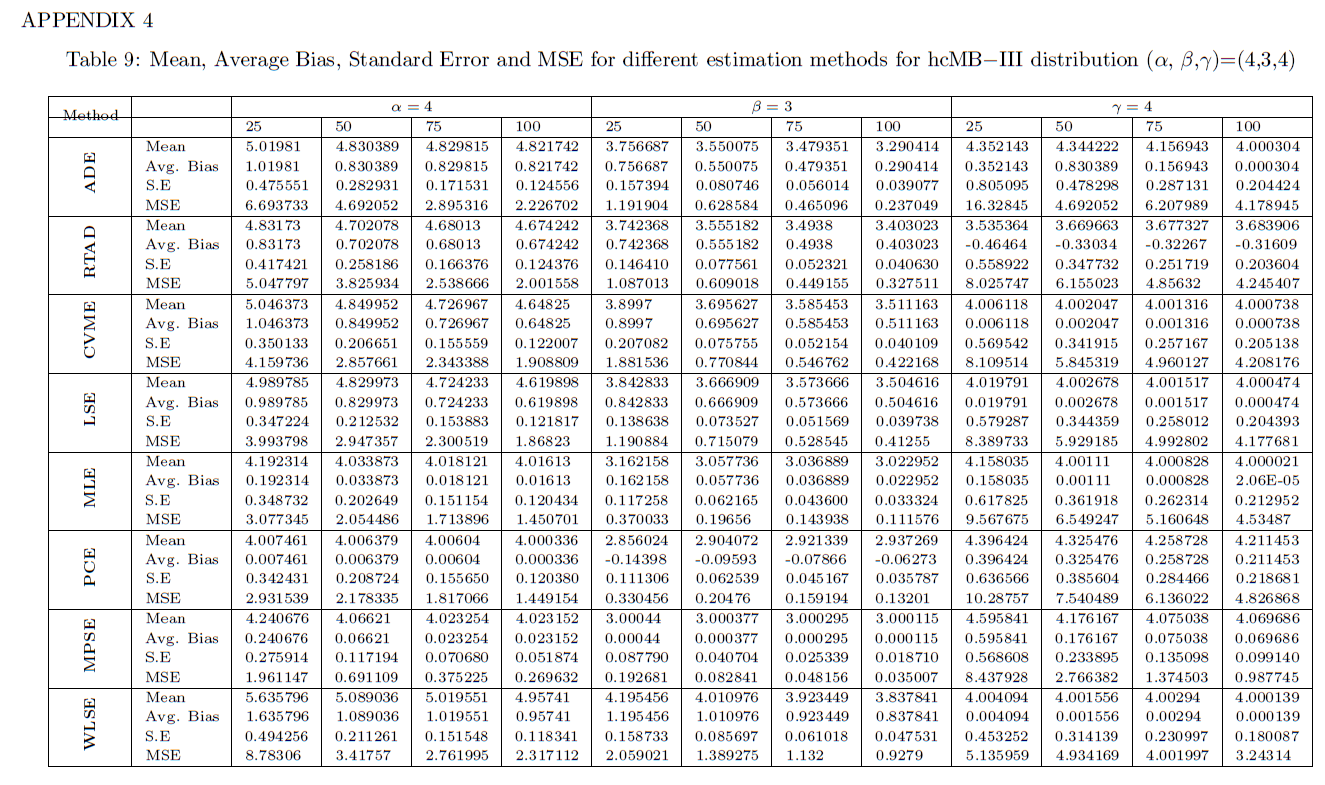

Supplement: S4 Appendix — (TIF) [file pone.0261901.s004.tif]
